# Differential expression of plasma exosomal microRNA in severe acute pancreatitis

**DOI:** 10.3389/fphar.2022.980930

**Published:** 2022-09-28

**Authors:** Yansong Xu, Yuansong Sun, Ran Yin, Tao Dong, Kai Song, Yang Fang, Guodong Liu, Bing Shen, He Li

**Affiliations:** ^1^ Department of Emergency, The Second Affiliated Hospital of Anhui Medical University, Hefei, Anhui, China; ^2^ School of Basic Medical Sciences, Anhui Medical University, Hefei, China; ^3^ Institute of Biomedical and Health Science, School of Life and Health Science, Anhui Science and Technology University, Chuzhou, Anhui, China

**Keywords:** severe acute pancreatitis, exosome, microRNA, target genes, biomarker, diagnosis

## Abstract

The incidence rate of acute pancreatitis is increasing, and severe acute pancreatitis (SAP) is associated with a high mortality rate, which may be reduced by a deeper understanding of its pathogenesis. In addition, an early determination of the severity of acute pancreatitis remains challenging. The aim of this study was to match potential biomarkers for early identification and monitoring of acute pancreatitis and to shed light on the underlying pathogenic mechanisms of SAP. The expression levels of plasma exosomal microRNA (miRNA) in patients with pancreatitis have been associated with the disease. Thus, this study compared the expression levels of exosomal miRNA in plasma collected from four patients with SAP and from four healthy participants. Analyses of the miRNA expression profiles indicated that three previously unreported miRNAs were differentially expressed in the patient group: Novel1, which was downregulated, and Novel2 and Novel3, which were upregulated. The miRNA target genes for those novel miRNAs were predicted using Metascape. Of these miRNA target genes, those that were also differentially expressed at different time points after disease induction in a mouse model of acute pancreatitis were determined. The gene for complement component 3 (C3), a target gene of Novel3, was the only gene matched in both the patient group and the mouse model. C3 appeared at most of the time points assessed after induction of acute pancreatitis in mice. These findings are foundational evidence that C3 warrants further study as an early biomarker of SAP, for investigating underlying pathogenic mechanisms of SAP, and as a therapeutic target for ameliorating the occurrence or development of SAP.

## Introduction

The rate of new cases of acute pancreatitis (AP), a serious threat to human health and life, is increasing (Banks et al., 2013). AP may be induced by cholelithiasis, excessive alcohol intake, hyperlipidemia, endoscopic retrograde cholangiopancreatography, and various drugs, all of which may cause pancreatin to leak into the pancreas, leading to inflammatory reactions, edema, hemorrhage, and even necrosis (Zhao et al., 2018; Stenwall et al., 2019). Damage to pancreatic tissue may lead to a clinical syndrome that includes acute upper abdominal pain, nausea, vomiting, fever, and increased blood levels of pancreatin. AP varies in severity. Moderate pancreatitis is characterized by edema of the pancreas and is typically a self-limiting disease with a good prognosis. Severe acute pancreatitis (SAP) often leads to pancreatic hemorrhage and necrosis with secondary infections, peritonitis, and shock and has a high mortality rate (Kitamura et al., 2017; Chatterjee et al., 2020). Recent clinical studies have shown that AP not only has an increased incidence rate but also a high mortality rate (Ji et al., 2016; Rijkers and van Eijck 2017; Vivian et al., 2019). During the last 50 years, as treatment for SAP has improved, cure rates have increased, but the pathogenesis associated with SAP is still not fully understood (Sandler et al., 2002; Fagenholz et al., 2007). In addition, it is challenging to make an early determination of the severity of AP. Therefore, it remains critical to find new and effective biomarkers for early identification and for monitoring AP.

Originally discovered in sheep reticulocytes in 1987, exosomes are round or oval vesicles with a diameter of approximately 30–120 nm and lipid membranes (Yáñez-Mó et al., 2015). They are released into the extracellular matrix after the fusion of intracellular multivesicular bodies with the cell membrane (Johnstone et al., 1987; Théry et al., 2002). Exosomes have been shown to play key roles in immunity, cellular homeostasis, autophagy, and cancer. (Pegtel and Gould 2019). Numerous studies have shown that exosomes contain microRNA (miRNA), small non-coding RNA present in human blood, urine, saliva, cerebrospinal fluid, and other body fluids (Bartel 2004; Chen et al., 2004; Bartel 2009). Exosomes may participate in the occurrence or development of diseases by transporting signal peptides, nucleic acids, lipids, and other biologically active molecules, especially by carrying miRNA, and play an important role in communication between cells (Simpson et al., 2009; Guiot et al., 2019; McVey et al., 2019; Ni et al., 2020). The miRNA may promote target gene degradation or inhibit target gene expression through post-transcriptional regulatory mechanisms. Importantly, exosomes have been used as biomarkers for the diagnosis and prognosis of diseases or even as clinical therapeutic targets (Meldolesi 2018; Lu et al., 2019; Manna et al., 2020). Thus, the expression level of plasma exosomal miRNA in patients with AP may provide important diagnostic or prognostic markers of this disease although, to date, this has been understudied.

Therefore, the present study used next-generation sequencing techniques to compare the plasma exosomal miRNA expression profile of patients with SAP with that of healthy participants. We used bioinformatics analyses to determine key miRNAs that were specific to patients with SAP, to predict target genes associated with these differentially expressed miRNAs, and to determine the key signaling pathways annotated with the target genes.

## Materials and methods

### Participants

Blood samples were collected from four patients with SAP (the patient group) admitted to the Emergency Department of the Second Affiliated Hospital of Anhui Medical University from March 2018 to August 2018 and from four healthy control participants (the control group) who visited the physical examination center of this hospital during that same time. Patients who met two of the following three criteria were regarded as having AP: 1) typical findings from computed tomography or abdominal ultrasonography; 2) characteristic symptoms with continuous abdominal pain; and 3) serum lipase or amylase levels that were higher than three times the normal reference upper limit. The Atlanta classification criteria revised in 2012 were used to define SAP as AP accompanied by continuous single or multiple organ dysfunction lasting at least 48 h (Banks et al., 2013). Patients with SAP who met all four of the following conditions were included: 1) admitted to the hospital within 24 h after onset; 2) received no treatment before admission and received treatment in our hospital after admission; 3) between 18 and 65 years of age; and 4) patient or guardian able to complete the investigation and provide all clinical data. Patients with SAP who met any of the following conditions were excluded: 1) previous history of AP; 2) failure to provide a specific time for the onset of sensory symptoms; 3) trauma history within 1 month; 4) having a malignant tumor; or 5) having a long history of hormone or immune-related drug use. The Medical Ethics Committee of the Second Affiliated Hospital of Anhui Medical University reviewed and approved our research protocol and an informed patient consent form. The study was conducted according to the principles of the Declaration of Helsinki, and written informed consent was obtained from the study participants prior to study commencement.

### Plasma exosome extraction

Blood samples were collected and centrifuged at 3,000 × g for 15 min to remove cells and cell debris. After transferring the supernatant to a sterile tube and adding an appropriate volume of ExoQuick Exosome Precipitation Solution (System Biosciences, Cat. No. EXOQ5A-1), we refrigerated the samples at 4 °C for at least 30 min. The samples were then centrifuged at 1,500 × g for 5 min. The supernatant was discarded and the precipitated exosomes were retained in the pellet.

### Nanoparticle tracking analysis

The freshly extracted exosomes were blown and mixed, and 10 μL was retained for detection. The exosomes were stained with the phosphotungstate (2%) negative staining technique, and the morphology of the exosomes was observed by transmission electron microscopy. Nanoparticle Tracking Analysis was used to determine the particle size distribution of the exosome samples. The exosome samples were also analyzed with Zetasizer Nano software.

### Western blot

Western blot analyses were used to detect the expression of the exosome surface marker proteins CD63 and TSG101. A radioimmunoprecipitation assay buffer was used to extract the exosomes. The proteins were separated on a sodium dodecyl sulfate polyacrylamide gel (10%) by using electrophoresis. The proteins were transferred to polyvinylidene fluoride membranes (Millipore, United States), which were blocked with 5% skimmed milk at room temperature for 2 h. Anti-CD63 (ab134045, Abcam, Britain) and anti-TSG101 (ab133586, Abcam, Britain) primary antibodies were added and incubated at 4 °C overnight. The next day, after three washouts using phosphate-buffered saline (PBS) with Tween 20, the horseradish peroxidase–conjugated secondary antibody (ab205718, Abcam, Britain) was added and the membrane was incubated at room temperature for 1 h. After washout, the proteins on the membrane were detected using an enhanced chemiluminescence detection system.

### Extraction of exosomal RNA

After exosomes were extracted by ultracentrifugation, the exosome precipitant was resuspended with 50 μL of PBS. We then added 700 μL of lysate to the samples and mixed well. The homogenate was incubated at room temperature for 5 min. After 140 μL of chloroform was added to mixture, the sample was shaken for 15 s, incubated at room temperature for 2–3 min, and centrifuged at 1.2 × 10^4^ *g* and 4 °C for 15 min. The upper aqueous phase was transferred to a new plastic tube, and anhydrous ethanol was added at 1.5 times the volume of the aqueous phase. Samples of 700 μL were placed on a filter column in a 2-ml collection tube, and the tube was centrifuged at room temperature (>8,000 × *g*) for 15 s to remove the fluid. Following washout with 80% ethanol, enzyme-free water was added to the filter column membrane, and it was centrifuged at 1.2 × 10^4^ *g* for 1 min to obtain RNA.

### Next-generation sequencing of miRNA and raw data quality control

A miRNA Sample PreKit was used to construct the library according to the instructions provided with the kit. The quality of the constructed library was tested and the qualified library was sequenced using a HiSeq 2,500 System.

Sequence quality control of the original sequence assessed using the sRNA library was conducted as follows: 1) for each sample, sequences with low quality values were removed; 2) reads with unknown base N (N is an unrecognized base) content greater than or equal to 10% were removed; 3) reads without a 3′ adaptor sequence were removed; 4) the 3′ joint sequence was cut off; 5) sequences shorter than 15 or longer than 35 nucleotides were removed. The sequencing data were submitted to the National Center for Biotechnology Information [NCBI]NCBI database (DATA number: PRJNA841245; Supplementary Table S1).

### Bioinformatics analysis

Cluster analysis was conducted using R language on the acquired miRNA expression profile to obtain information about the miRNAs and their sequences. The top 20 target genes of the miRNAs matched in our study were analyzed by Gene Ontology (GO) and Kyoto Encyclopedia of Genes and Genomes (KEGG) enrichment analyses. To find genes that were potentially specifically associated with SAP, we determined the target genes of the differentially expressed miRNAs found in our study that were common to the results found by other group who assessed the RNA sequence profile in a mouse model of AP and uploaded their results as transcription profile GSE65146 to the National Center for Biotechnology Information GEO database (http://www.ncbi.nlm.nih.gov/geo/). The authors of that study used consecutive abdominal injections of caerulein to induce AP. Caerulein or a control salt solution was injected at 0 h, and tissue samples were collected at 0, 3, 12, 24, 36, 48, 60, 72, 84, 96 h, 5, 7, and 14 days. Each time point had three mice. After the mice were killed, pancreatic total tissues were collected and Affymetrix GeneChip Mouse Gene 1.0 ST arrays were used for RNA sequencing. We matched the differentially expressed genes (DEGs) in these wild-type mice by using GEO2R (*p <* 0.05, Log_2_ fold change absolute value > 1). We then found the target genes of the miRNAs matched in our study that intersected with the DEGs obtained at different time points in the mouse model of AP.

### Statistical analysis

Measurement and count data obtained from the study participants are showed as means ± standard deviations or as percentages, respectively. We used SPSS, version 23.0 (SPSS Inc.) and R (version, 4.0.3) software to perform statistical analyses. Comparisons between two groups were performed using the Fisher exact test and unpaired Student’s *t*-test. Two-tailed *p* values <0.05 were considered statistically significant.

## Results

### Demographic and clinical characteristics of participants

In total, eight participants were enrolled in this study, including four healthy volunteers and four patients with SAP. The baseline characteristics of these eight participants are given in Table 1. The fasting blood glucose (patient group, 12.25 ± 3.08 vs. control group, 5.06 ± 0.52 mmol/L; *n* = 8, *p* = 0.017), total cholesterol (patient group, 9.97 ± 1.64 vs. control group, 4.81 ± 0.86 mmol/L; n = 8, *p* = 0.001), triglycerides (patient group, 3.47 ± 0.56 vs. control group, 1.15 ± 0.22 mmol/L; n = 8, *p* < 0.001), white blood cell (patient group, 15.06 ± 2.15 × 10^9^/L vs. control group, 6.52 ± 1.29 × 10^9^/L; n = 8, *p* < 0.001), and amylase (patient group, 723.25 ± 49.03 vs. control group, 58.50 ± 5.00 U/L; n = 8, *p* < 0.001) were significantly lower in the control group than in the patient group (Table 1).

**TABLE 1:** Demographic and clinical characteristics of patients with severe acute pancreatitis and of healthy participants.

Characteristic	Severe acute pancreatitis (n = 4)	Healthy participants (*n* = 4)	Statistical Result	*p* Value
Sex, (%)				1.000^a^
Male	2 (50)	2 (50)		
Female	2 (50)	2 (50)		
Smoker (%)	2 (50)	1 (25)		1.000^a^
Alcoholism (%)	1 (25)	1 (25)		1.000^a^
Age (years)	52.00 ± 4.55	46.50 ± 8.10	1.184	0.281^b^
BMI (kg/m^2^)	25.05 ± 4.21	21.74 ± 1.96	1.426	0.204^b^
SBP (mmHg)	135.25 ± 3.77	118.50 ± 11.33	2.806	0.054^b^
DBP (mmHg)	86.50 ± 5.57	79.25 ± 8.73	1.400	0.211^b^
FBG (mmol/L)	12.25 ± 3.08	5.06 ± 0.52	4.610	0.017^b^
TC (mmol/L)	9.97 ± 1.64	4.81 ± 0.86	5.578	0.001^b^
TG (mmol/L)	3.47 ± 0.56	1.15 ± 0.22	7.685	<0.001^b^
WBC (×10^9^/L)	15.06 ± 2.15	6.52 ± 1.29	6.816	<0.001^b^
Amylase (U/L)	723.25 ± 49.03	58.50 ± 5.00	26.974	<0.001^b^

BMI, body mass index; SBP, systolic blood pressure; DBP, diastolic blood pressure; FBG, fasting blood glucose; TC, total cholesterol; TG, triglycerides; WBC, white blood cell. The *p* value was obtained for comparison of the groups with following tests: ^a^Fisher exact test; ^b^Unpaired Student’s *t*-test.

### Identification of exosomes

**FIGURE 1:**
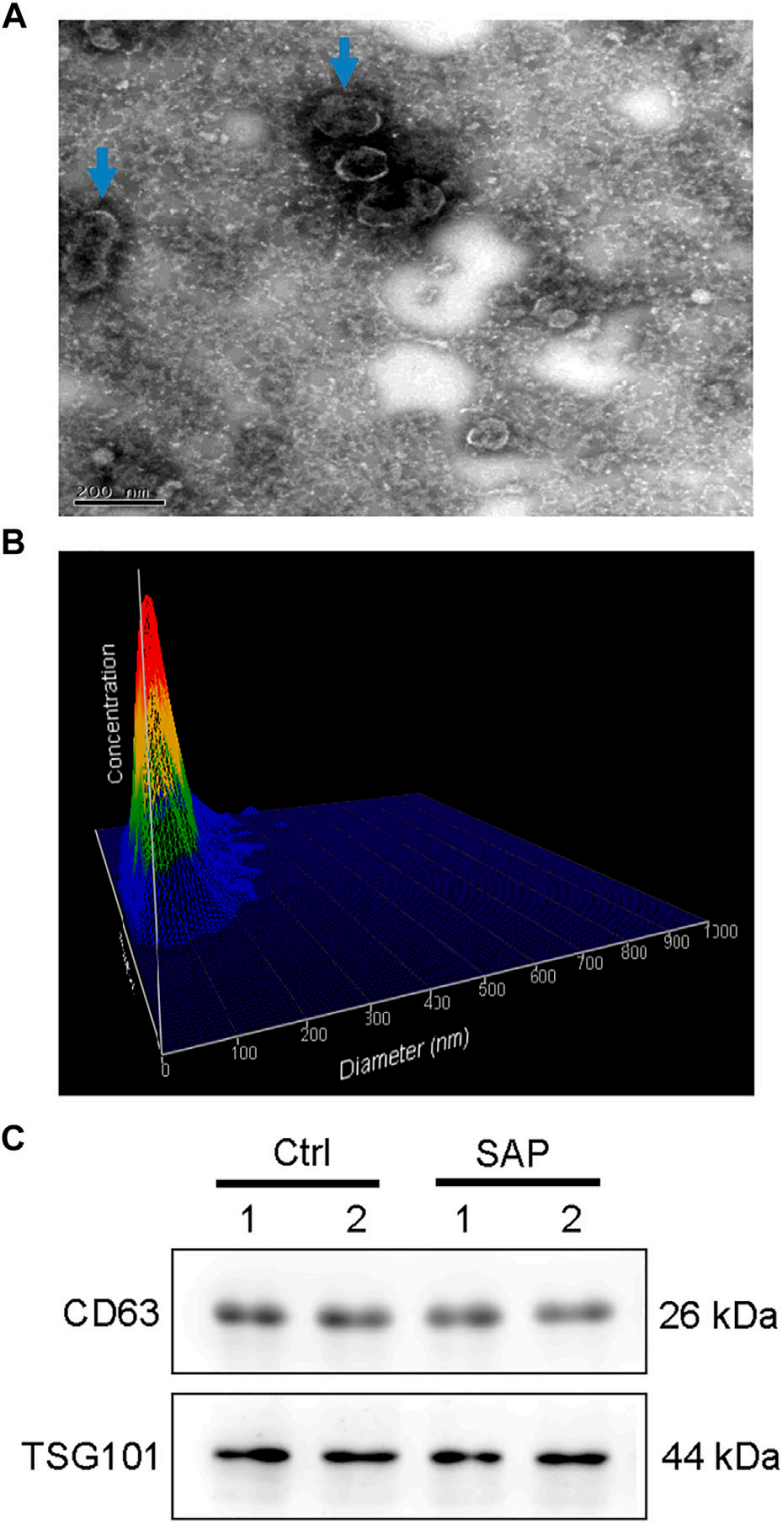
Plasma exosome identification **(A)** Morphology of plasma exosomes. Image captured using transmission electron microscopy. Arrows point to exosomes. **(B)** Plasma exosomes size distribution by volume **(C)** Representative images showing the expression of the exosome-specific surface markers CD63 and TSG101 in exosomes extracted from the plasma of patients with severe acute pancreatitis (SAP) and healthy control (Ctrl) participants.

The exosomes extracted from the plasma samples of participants were observed by transmission electron microscopy. The exosomes appeared as saucer-shaped microvesicles (Figure 1A, arrows). The Nanoparticle Tracking Analysis results indicated that the diameters of the exosomes ranged from 40 to 150 nm, with a peak of 80 nm (Figure 1B). To confirm that the microvesicles were exosomes, we used Western blotting to match protein biomarkers specific to exosomes. Figure 1C shows that the microvesicles expressed the exosome-specific surface marker proteins TSG101 and CD63. Thus, our extraction of exosomes from the plasma of patients with SAP and from healthy control participants was successful.

### Quality of exosomal miRNA sequencing data

**FIGURE 2:**
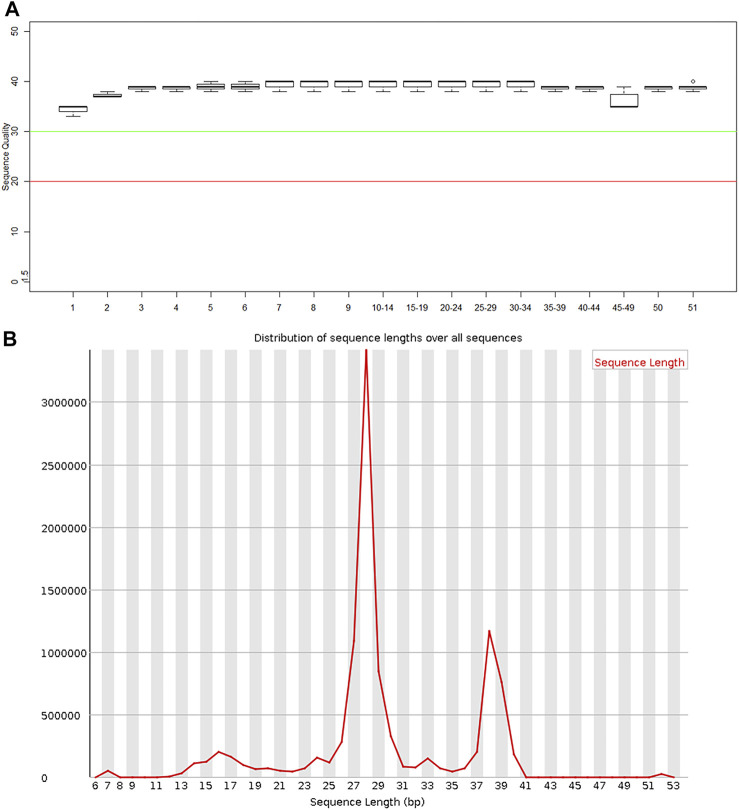
Quality control results of miRNA in plasma exosomes **(A)** Sequence quality of the plasma exosomal miRNA extracted from patients with severe acute pancreatitis. **(B)** Read length distribution of the plasma exosomal miRNA sequences extracted from patients with severe acute pancreatitis.

FastQC software was used to perform quality control checks on the obtained forward sequences. As shown in Figure 2A, all sequences scored above the Q30 line, that is, they all had a sequence quality score of 30 or higher, indicating that the data quality was sufficient for use in this study. In addition, the sequence lengths were mostly distributed around 28 bp (Figure 2B), consistent with the size of miRNA.

### Comparison of plasma exosomal miRNA between patients with SAP and healthy control participants

**FIGURE 3:**
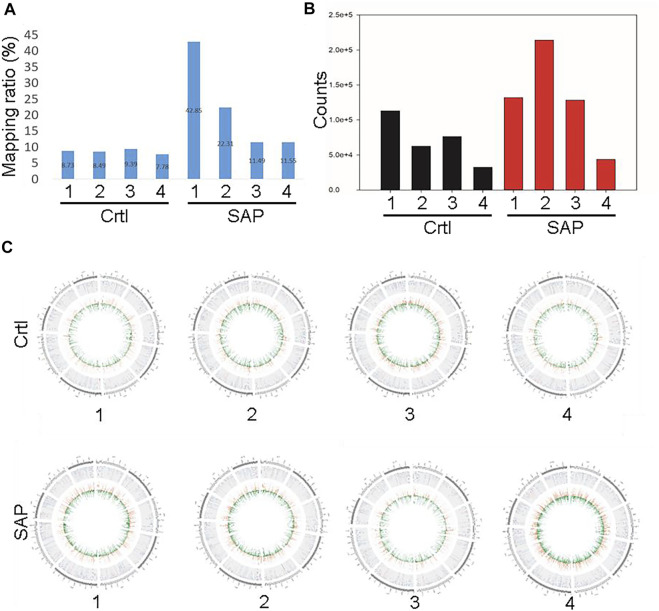
Comparison of plasma exosomal miRNA extracted from patients with severe acute pancreatitis (SAP) vs. healthy controls (Ctrl) **(A)** Mapping ratio of the plasma exosomes and **(B)** total plasma exosomal miRNA expression in patients with SAP and healthy controls (Ctrl). **(C)** Distribution density of reads in each chromosome. Numbers one to four refer to the patient or participant identification number.

The exosomal miRNA sequence data obtained from patients with SAP and from control participants were compared with the human reference genome HG19. Figure 3A shows the percentages of the whole reference genome of HG19 that were miRNA sequences from patients with SAP and from control participants. The mean percentage of the reference genome that was exosomal miRNA extracted from four patients with SAP was 22.05 ± 14.77%, whereas the mean expression level extracted from four healthy participants was much lower, only 8.60 ± 0.66% (Figure 3A). When the total miRNA expression levels in each sample were compared, we found that the expression levels of plasma exosomal miRNA were increased in patients with SAP compared with healthy controls (Figure 3B). All samples were compared to reads on each chromosome of the genome for density statistics, and Circos was used to draw a map to check the distribution of reads on each chromosome (Figure 3C).

### Cluster analysis of exosomal miRNA

**FIGURE 4:**
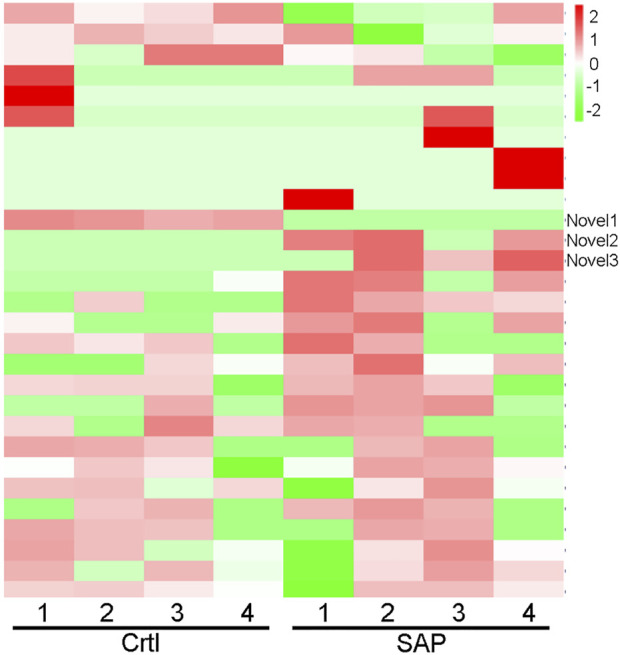
Cluster analysis of miRNA expression profile. Cluster analysis data showing the expression levels of plasma exosomal miRNA in patients with severe acute pancreatitis (SAP) and healthy control (Ctrl) participants. Novel1, Novel2, and Novel3 are indicated. Red represents high level of expression; green, low level of expression.

**FIGURE 5:**
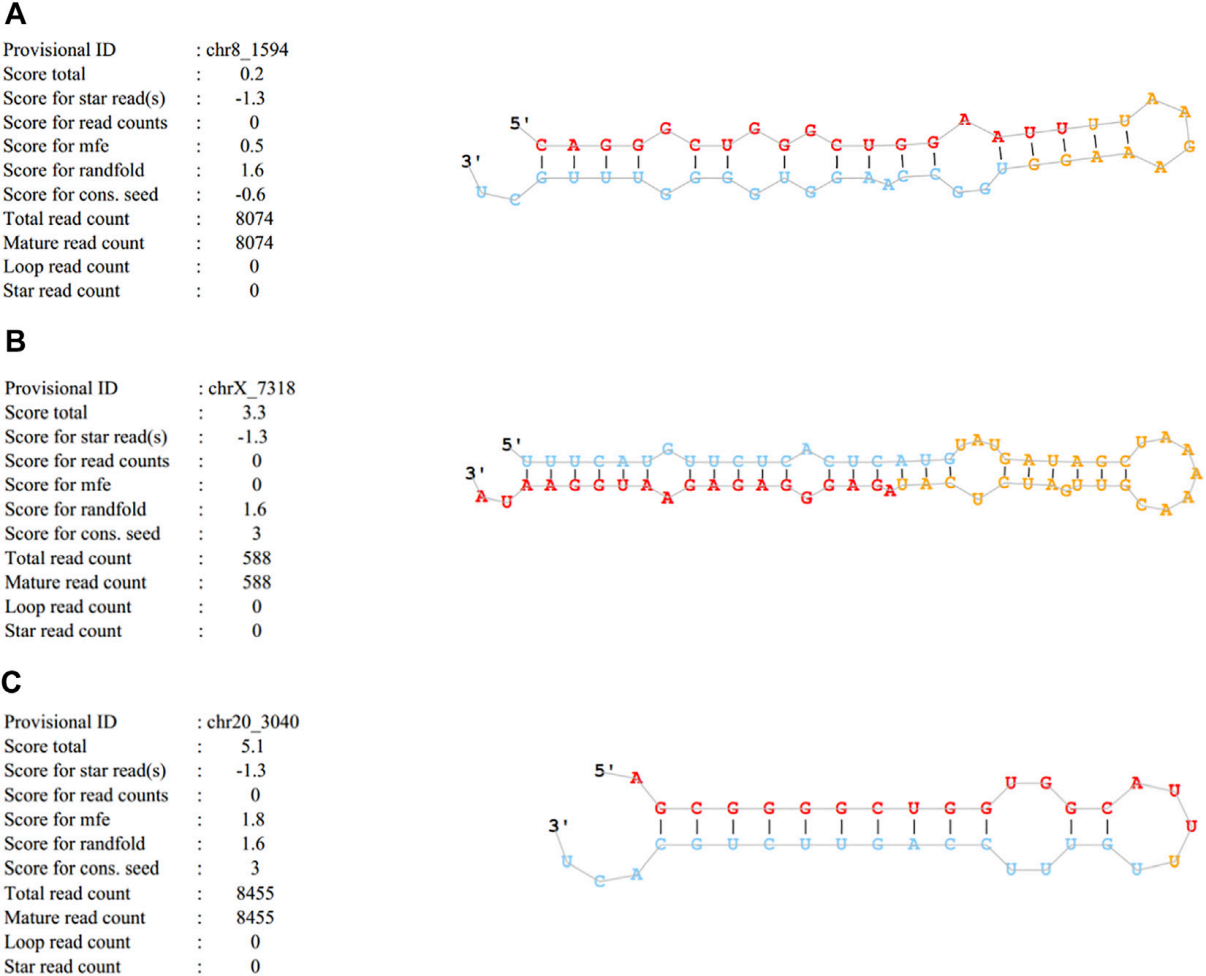
Basic information and sequence structures of three novel miRNAs **(A)** Novel1, **(B)** Novel2 and **(C)** Novel3.

Cluster analysis showing exosomal miRNA expression profiles was performed using R language (Figure 4). Obvious differences were observed in the profiles between patients with SAP and healthy controls. In addition, one novel miRNA, termed Novel1, was significantly downregulated, and two novel miRNAs, termed Novel2 and Novel3, were significantly upregulated in patients with SAP compared with healthy controls (Supplementary Table S2). The mature sequences and precursor sequences are provided in Supplementary Table S2. Additional information and sequence structures of three novel miRNAs are shown in Figure 5.

### Signaling pathway analysis of miRNAs target genes

**FIGURE 6:**
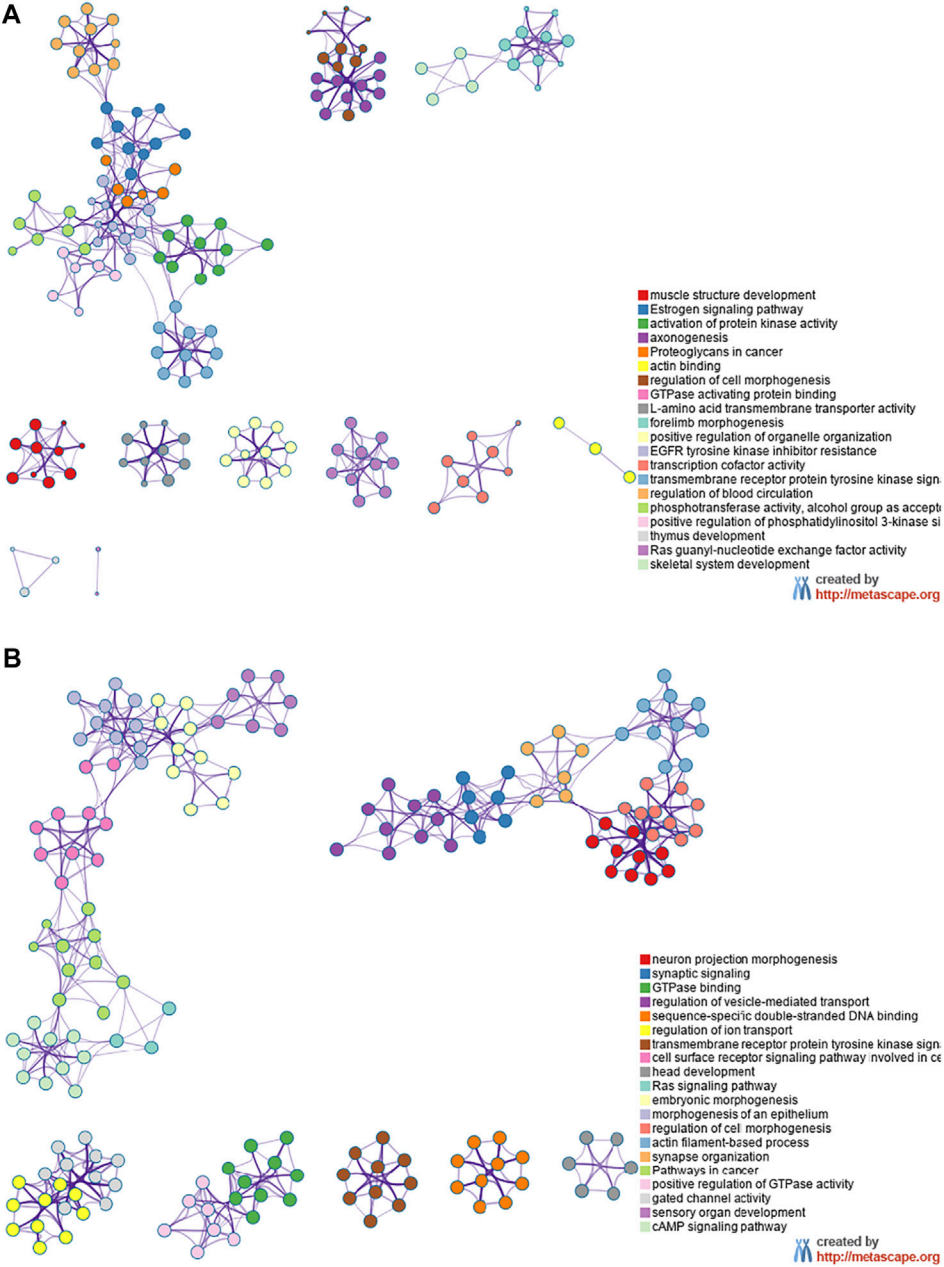
Kyoto Encyclopedia of Genes and Genomes and Gene Ontology analyses. Data showing downregulated **(A)** and upregulated **(B)** miRNA target genes in plasma exosomes extracted from patients with severe acute pancreatitis, compared with healthy control participants.

The miRNA target genes were predicted using the online tool Metascape. The downregulated miRNA Novel1 had 1,549 predicted target genes, the upregulated miRNA Novel2 had 2,431 predicted target genes, and the upregulated miRNA Novel3 had 1,549 predicted target genes. The target genes of these three novel miRNAs were analyzed using KEGG and GO, and the top 20 signaling pathways associated with the target genes are shown in Figure 6. As shown in Figure 6A, numerous Novel1 target genes were involved in the estrogen signaling pathway, activation of protein kinase activity, and EGFR tyrosine kinase inhibitor resistance. In addition, numerous target genes of Novel2 and Novel3 were involved in morphogenesis of an epithelium, regulation of vesicle-mediated transport, and embryonic morphogenesis (Figure 6B).

### DEGs in pancreatic tissue of a mouse model of AP that intersect with target genes of novel plasma exosomal miRNAs from patients with SAP

To find genes that may play potentially key roles in SAP, we downloaded from the National Center for Biotechnology Information website an RNA sequence dataset from a mouse model of AP. We used GEO2R to find DEGs in the pancreatic tissues of the mouse model at each time point following an injection of caerulein to induce AP (Supplementary Figure S1). We then compared these DEGs with the target genes of Novel1, Novel2 and Novel3 to match those that were common to both. The results indicated that one target gene of Novel3, the gene for complement component 3 (C3), intersected with the DEGs in the mouse model of AP at 3, 24, 36, 48, 60, 72, 84, 96 h, 5 days and 7 days (Supplementary Figure S2). This finding suggested that C3 may be a key gene involved in AP.

## Discussion

In this study, plasma samples were collected from four patients with SAP and from four healthy participants, and a large number of miRNAs were detected in the extracted exosomes. Compared with those in the healthy control group, three differentially expressed miRNAs were matched in the exosomes of patients with SAP, namely Novel1, which was downregulated, and Novel2 and Novel3, which were upregulated. After comparing the predicted target genes of miRNA Novel1, Novel2, and Novel3 with the genes differentially expressed at various times after induction of a mouse model of AP (Kong et al., 2016), we found that one miRNA Novel3 target gene, C3, intersected with the mouse DEGs at nearly every time point.

From a pathophysiological point of view, the occurrence and development of mild AP (MAP) and SAP have common characteristics involving recruitment and activation of neutrophils, production of various cytokines and vasoactive substances, and activation of related signaling pathways; however, MAP and SAP are diametrically opposite in their courses and severity (Weber and Adler 2001; Makhija and Kingsnorth 2002). MAP is not typically associated with local or systematic severe complications but accompanies a milder disease course, with most patients recovering spontaneously (Beger et al., 1997). By contrast, more than 20% of AP may gradually worsen to SAP, with patients experiencing severe complications, such as pancreatic necrosis, systemic inflammatory response syndrome, and even multiple organ dysfunction syndrome (Garg et al., 2005), resulting in an overall mortality rate of approximately 40%. Although some researchers have attempted to describe the pathogenesis of AP based on changes in proteins and signaling pathways (Shields et al., 2002; Gukovsky et al., 2004; Pereda et al., 2004), the pathogenesis remains unclear owing to the complexity of the disease.

Increasingly more attention is being paid to the biological functions of exosomes. For example, exosomes have been suggested as biomarkers for the diagnosis and prognosis of diseases and even as clinical therapeutic targets (Meldolesi 2018; Lu et al., 2019; Manna et al., 2020). The present study focused on changes in the miRNA expression profile of plasma exosomes in patients with SAP and the association of these changes with the pathogenesis of SAP. Through next-generation sequencing, three differentially expressed miRNAs were found, none of which has been previously reported. We compared the target genes of the three differentially expressed miRNAs, matched by sequencing, with the differentially expressed genes matched in the GSE65146 sequencing data from the mouse model of AP published in an existing database (Kong et al., 2016). We found one common gene: C3.

C3 is a 185-kDa glycoprotein encoded by the C3 gene and consists of a 110-kDa α chain and a 75-kDa β chain (de Bruijn and Fey 1985). C3, synthesized primarily by liver cells and distributed in blood vessels (Morgan and Gasque 1997), is one of the most abundant plasma proteins in the circulation. Its high plasma levels and low biological reactivity enable it to act as a sentinel in its natural state such that it can quickly respond as an acute phase reactant in the face of potential threats. C3 participates in all three complement pathways by cleavage into two active components: C3a and C3b (Asgari et al., 2013). C3b binds to complement receptor 1 (CR1, CD53) to increase the conditioning effect of the immune complex. At present, there are few studies on the function of the complement system in AP (Linders et al., 2020). In a mouse model, Linders et al. (Linders et al., 2020) demonstrated that C3 induces neutrophil aggregation and plays a critical role in ensuring the formation of neutrophil extracellular traps. Neutrophil extracellular traps induce pancreatic tissue inflammation by triggering trypsinogen activation (Merza et al., 2015). Therefore, C3 may warrant further study as an important target for ameliorating local pancreatic injury. Several reports have confirmed the detection of low levels of C3 in the plasma of patients with AP (Whicher et al., 1982; Ueda et al., 2006; Zhang et al., 2020). Further evidence suggests that C3 is even more sensitive than C-reactive protein, a general inflammatory marker, for the early prediction of SAP (Zhang et al., 2020). In addition, low levels of C3 are considered to be an adverse prognostic factor for SAP (Ueda et al., 2006). However, a few studies have suggested that C3 is not correlated with AP, that the low level of C3 is only due to trypsin digestion (Whicher et al., 1982). By contrast, more studies have indicated that trypsin cleaves and activates the central complement component, leading to the formation of C3a, suggesting that complement may indeed be involved in the development of AP (Minta et al., 1977). Moreover, Balldin et al. have shown that catabolism of complement occurs primarily in the abdominal cavity and is the result of a protease-antiprotease imbalance (Balldin et al., 1981). Another study also provides evidence supporting the idea that C3a levels are associated with disease severity and are good predictors of AP (Gloor et al., 2003).

In addition to its role in AP, C3 is involved in other diseases, especially in immune diseases (Lintner et al., 2016; Kim et al., 2019; Smith et al., 2019). The pathology of systemic lupus erythematosus is a highly complement-dependent process, and plasma concentrations of C3 and its lysates can be used as biomarkers of systemic lupus erythematosus disease activity (Atkinson 1986; Kim et al., 2019). A large number of studies have shown that C3 and its lysates may promote the occurrence and development of age-related macular degeneration. Although the specific mechanism underpinning its role in this degeneration is not clear, evidence at the gene level (Yates et al., 2007) and plasma test results (Maller et al., 2007) agree with its involvement. In addition, C3 is involved in the occurrence and development of Alzheimer disease. The main pathological feature of Alzheimer disease is the aggregation of neurofibrillary tangles caused by hyperphosphorylation of Tau protein (Rafii 2016; Bejanin et al., 2017). C3a receptor antagonists have been found to significantly reduce the phosphorylation of Tau at Ser396 and Thr231 (Shi et al., 2015; Hu et al., 2019). This evidence suggests that the complement system is a promising therapeutic target for Alzheimer disease. In addition, complement fragments C3a and C5a regulate the transport of normal hematopoietic cells but have no effect on cell proliferation and survival (Lenkiewicz et al., 2019). Unfortunately, this physiological effect also enhances the ability of malignant cells in patients with leukemia/lymphoma to move and thus to spread (Wysoczynski et al., 2015; Abdelbaset-Ismail et al., 2017). Complement proteins affect not only the locomotion ability of hematopoietic cells but also the phenotype of macrophages (Phieler et al., 2013; Ruan et al., 2015). In a mouse model of unilateral ureteral obstruction, Cui et al. (Cui et al., 2019) found that C3 knockout reduces M1 macrophages in the kidney but increases M2 macrophages. M1 macrophages are thought to aggravate cell damage, whereas M2 macrophages are involved in cell repair (Lee et al., 2011; You et al., 2013; Zhang et al., 2017). In addition to the circulating C3 produced mainly by the liver, local C3 produced by other tissues also plays important roles in physiological processes of the body (Tang et al., 1999; Fisher et al., 2017). For example, locally synthesized C3 promotes renal fibrosis via the C3a receptor in the mouse model of unilateral ureteral obstruction (Xavier et al., 2017; Liu et al., 2018). Although C3 is important in regulating the functional expression of neutrophils and macrophages, the specific regulatory mechanisms of C3 in AP were neither investigated nor clarified in this study; this is the direction of our future research.

In summary, **t**his study compared plasma exosomal miRNA profiles extracted from patients with AP and from healthy participants and found three differentially expressed miRNAs. By using robust prediction and comparison analyses, we determined that one of the predicted miRNA target genes, C3, was associated with AP. In addition, although this study found some differentially expressed miRNAs through rigorous methods, there were still some limitations. The sample size of this research was small. Secondly, the suboptimal sample size may increase the bias of some parameters, such as gender. Finally, we have not clarified the mechanism of this differentially expressed miRNAs and target gene C3 on AP in this study. In the future, we can show via gain- and/or loss-of-function studies that Novel1,2, and/or three biologically regulate mRNA levels of C3 in any immune cell or parenchymal cell relevant to SAP.

## Data Availability

The datasets presented in this study can be found in online repositories. The names of the repository/repositories and accession number(s) can be found in the article/Supplementary Material.
