# Supplementary material for: Differential expression of plasma exosomal microRNA in severe acute pancreatitis
Source: Front Pharmacol. 2022 Sep 28;13:980930. doi: 10.3389/fphar.2022.980930 (PMC9554001; doi:10.3389/fphar.2022.980930)
Supplement: Supplementary file 1 [file Table1.DOCX]

Supplementary Material

**Supplementary Table 1.** Raw sequencing data of plasma exosomal microRNA from all participants in this study.

The datasets [sequencing data] for this study can be found in the [National Center for Biotechnology Information, NCBI] [https://www.ncbi.nlm.nih.gov/bioproject/PRJNA841245].

**Supplementary Table 2.** Novel miRNAs differentially expressed in plasma exosomes extracted from patients with severe acute pancreatitis.

| **miRNA** | **Log FC** | ***P* value** | **miRDeep2 score** | **Consensus mature sequence** | **Consensus precursor sequence** | **Precursor coordinate** |
| --- | --- | --- | --- | --- | --- | --- |
| Novel1 | -15.25 | < 0.01 | 0.2 | cagggcugggcuggaauu | cagggcugggcuggaauuuuaagaaagguggccaaggugggguuugcu | chr8:143786724..143786772:+ |
| Novel2 | 12.54 | < 0.01 | 3.3 | agagggagagaauggaaua | uuucauguucucacucauguaugauagcuaaaaacguugaucucauagagggagagaauggaaua | chrX:135312181..135312246:- |
| Novel3 | 12.52 | 0.01 | 5.1 | agcggggcugguggcauu | agcggggcugguggcauuuuguuuccaguucugcacu | chr20:46800508..46800545:- |

FC, fold change.


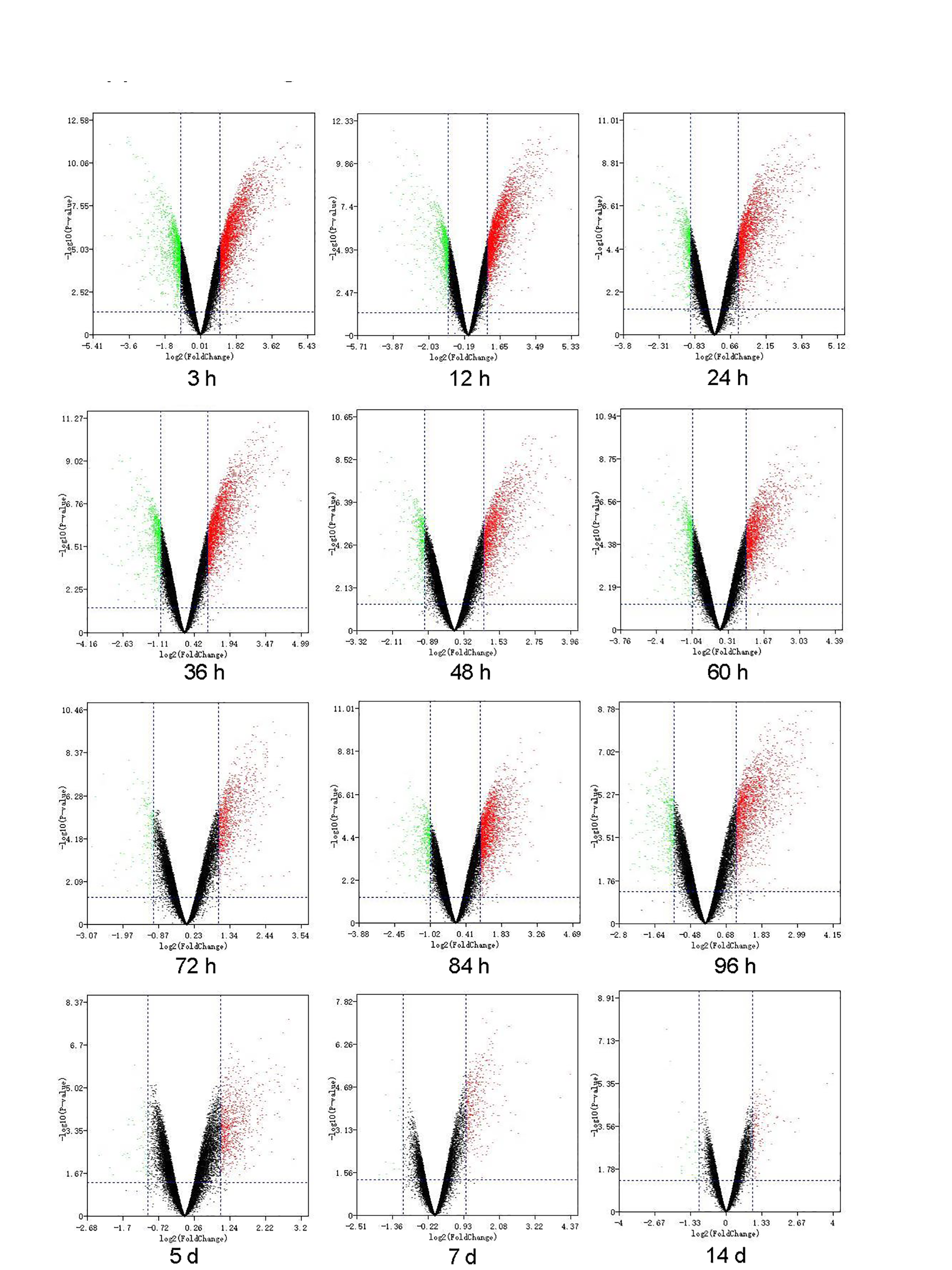
**Supplementary Figure 1.** Differentially expressed genes in pancreatic tissues of a mouse model of acute pancreatitis at different time points after disease induction. Time points indicate the time after injections of caerulein to induce acute pancreatitis.


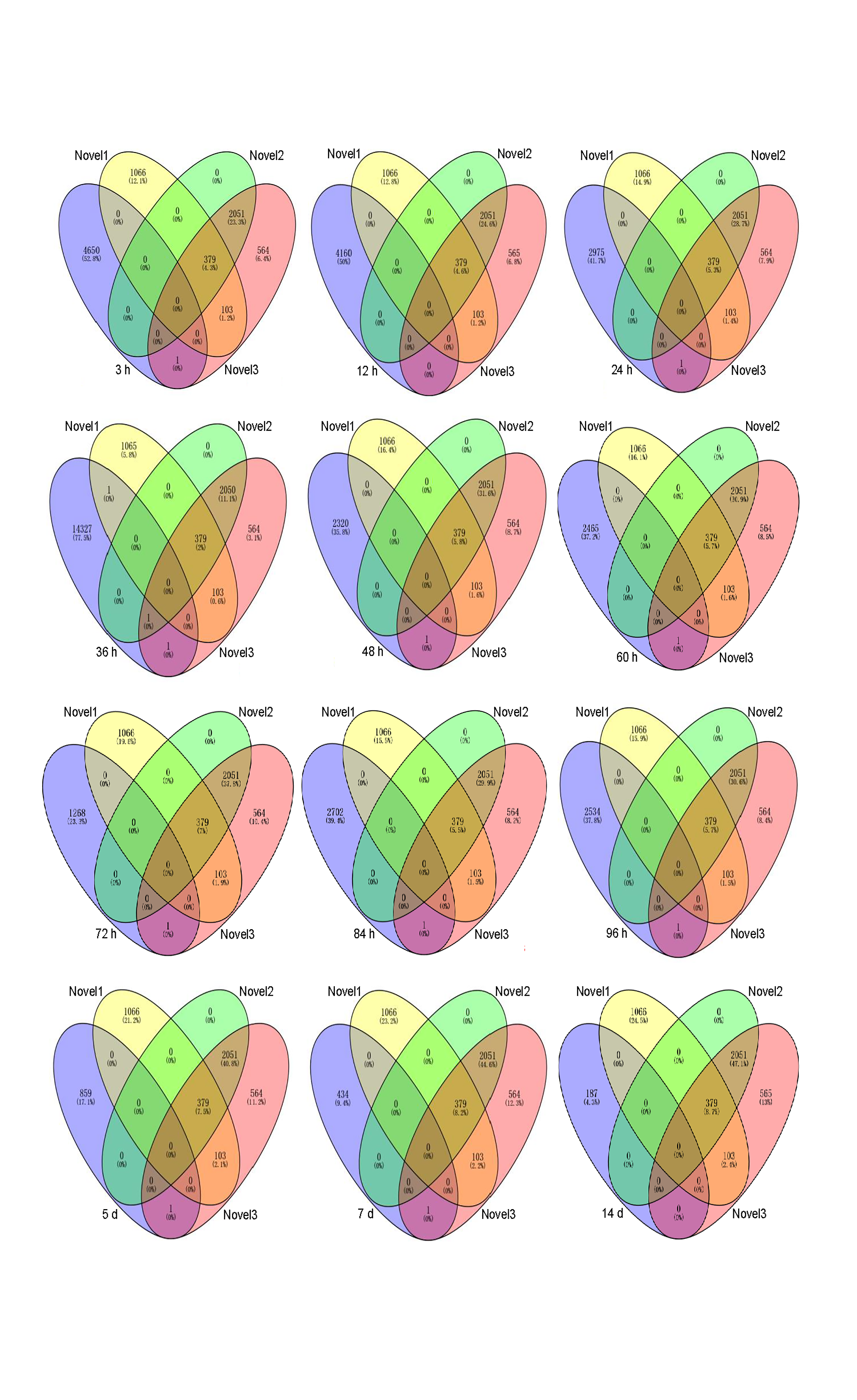


**Supplementary Figure 2.** Intersections of genes differentially expressed in pancreatic tissue from a mouse model of acute pancreatitis and target genes of novel plasma exosomal miRNAs from patients with severe acute pancreatitis. Venn diagrams showing the intersections of differentially expressed genes at different time points after disease induction in the mouse model of acute pancreatitis with miRNA Novel1, Novel2, and Novel3 target genes.
